# A review of pediatric fasting guidelines and strategies to help children manage preoperative fasting

**DOI:** 10.1111/pan.14738

**Published:** 2023-08-02

**Authors:** Eileen Zhang, Neil Hauser, Aine Sommerfield, David Sommerfield, Britta S. von Ungern‐Sternberg

**Affiliations:** ^1^ Department of Anaesthesia and Pain Medicine Perth Children's Hospital Perth Western Australia Australia; ^2^ Perioperative Medicine Team, Perioperative Care Program Telethon Kids Institute Perth Western Australia Australia; ^3^ Division of Emergency Medicine, Anaesthesia and Pain Medicine The University of Western Australia Perth Western Australia Australia

**Keywords:** anesthesia, children, fasting, pediatric, tolerance

## Abstract

Fasting for surgery is a routine step in the preoperative preparation for surgery. There have however been increasing concerns with regard to the high incidence of prolonged fasting in children, and the subsequent psycho‐social distress and physiological consequences that this poses. Additionally, the past few years have yielded new research that has shown significant inter‐individual variation in gastric emptying regardless of the length of the fast, with some patients still having residual gastric contents even after prolonged fasts. Additionally, multiple large‐scale studies have shown no long‐term sequalae from clear fluid aspiration, although two deaths from aspiration have been reported within the large Wake Up Safe cohort. This has led to a change in the recommended clear fluid fasting times in multiple international pediatric societies; similarly, many societies continue to recommend traditional fasting times. Multiple fasting strategies exist in the literature, though these have mostly been studied and implemented in the adult population. This review hopes to summarize the recent updates in fasting guidelines, discuss the issues surrounding prolonged fasting, and explore potential tolerance strategies for children.

Reflective questions

- What are the fasting protocols at your institution, and how do these compare with the most recent evidence and various guideline updates?
- What are the negative physiological and psychological effects of prolonged fasting in children?
- What are the gastric emptying times in children?
- Are there any tolerance strategies in place at your institution?
- Do you have a problem—do know the incidence of prolonged preoperative fasting at your hospital?

## 1 INTRODUCTION

A cornerstone of preoperative preparation for anesthesia is the fasting of patients. This may be particularly distressing for young children and lead to a range of negative psycho‐social as well as physiological outcomes.^1^, ^2^, ^3^, ^4^, ^5^, ^6^, ^7^, ^8^ Fasting before general anesthesia reduces the volume of gastric contents in an effort to reduce the risk of gastric regurgitation and the consequences of any pulmonary aspiration.^1^ “Fasting from midnight” made its appearance in the 1970s, after multiple case reports in adults of pulmonary aspiration during anesthesia.^1^ This dogma was subsequently adopted around the world, in both the adult and pediatric populations, as common practice. While pulmonary aspiration is the commonest cause of death during anesthesia in adults,^9^ to date there have been very few pediatric deaths, or long‐term sequalae, reported from aspiration in the pediatric population.^10^, ^11^, ^12^

In recent times, there have been considerable efforts to find a better balance between sufficient fasting times to avoid aspiration, while simultaneously reducing the negative physiological outcomes and stress on our young patients. This is also in line with consumer priorities for perioperative medicine.^13^ Indeed, a study of children undergoing emergency surgery found the most common negative recollection was fasting, together with pain.^14^ It therefore seems prudent to find strategies to improve fasting tolerance in children. The scope of this review is to explore the updates in recent fasting guidelines and examine the adverse effects of prolonged fasting in children and the proposed solutions.

Pediatric fasting guidelines are typically divided into clear fluids, breast milk, formula, and solids. Clear fluids have been defined by the Australian and New Zealand College of Anaesthetists (ANZCA) as “water, carbohydrate rich fluids, specifically developed for perioperative use, pulp free fruit juice, clear cordial, black tea and coffee … excludes fluids containing particulate matter, soluble fibre, milk‐based drinks and jelly.”^15^ Internationally, solids have also been further divided into light breakfasts (specified as “buttered toast with jam or cereals with milk”^16^) and solids (other foods not previously included).^17^

The pediatric fasting guidelines have recently been updated in multiple countries (Table 1) to reflect the latest evidence for gastric emptying, and the minimal morbidity and mortality data reflected in pediatric patients who do aspirate. Of note, ANZCA, the Association of Paediatric Anaesthetists of Great Britain and Ireland, the European Society of Anaesthesiology and Intensive Care, L'Association Des Anesthésistes‐Réanimateurs Pédiatriques d'Expression Française, and the Canadian Anesthesiologists' Society have all reduced their recommended fasting time for clear fluids to 1 h, down from the traditional 2 h.^15^, ^16^, ^18^, ^19^ Indeed, the latest guideline from the ESAIC has recommended the avoidance of prolonged fasting times in all children where possible, with the encouragement of clear fluids up to 1 h pre‐induction for healthy children having elective surgery.^16^ This has not been reflected by the American Society of Anesthesiologists.^20^, ^21^ It remains however up to the discretion of each institution to set their fasting protocols, based on national guidelines, leading to the inter‐hospital variation seen in practice.^22^, ^23^, ^24^ Additionally, the individual patient should be taken into account in the context of pathologies that result in delays to gastric motility.^16^

**TABLE 1:** Pediatric fasting guidelines of various Anesthesia Societies.

Institution	Clear fluids	Breast milk	Nonhuman milk including formula	Solids	Chewing gum	Year updated
ANZCA^15^						
Children <6 month	1 h	3 h	4 h	–	Nil formal (discard for aspiration risk)	2017
Children >6 month	1 h (max 3 mL/kg/h)	4 h	4 h	6 h		
APABGI (no age differentiation)^19^, ^25^	1 h	4 h	6 h	6 h	2 h	2018
ESAIC^16^						
Infants	1 h	3 h	4 h	–	–	2022
General	1 h	–	–	4 h (light breakfast or nonclear fluid) 6 h solids	Nil formal (discard prior to induction)	
ASA (no age differentiation)^20^, ^21^	2 h	4 h	6 h	6 h (light meal). Consider further fasting for fried/fatty foods or meat	Nil for pediatrics	2023
CAS (no age differentials)^18^	1 h	4 h	6 h	6 h (light meal) 8 h (large solids, especially protein or fatty foods)	Nil	2021

Abbreviations: ANZCA, Australian and New Zealand College of Anaesthetists; APAGBI, Association of Paediatric Anaesthetists of Great Britain and Ireland; ASA, American Society of Anesthesiologists; CAS, Canadian Anesthesiologists' Society; ESAIC, European Society of Anaesthesiology and Intensive Care.

## 2 THE INTER‐INDIVIDUAL VARIATION OF GASTRIC EMPTYING

Despite the precautions with fasting times, recent research has shown significant inter‐individual variation in gastric emptying regardless of the length of the fast undertaken by pediatric patients. While having a large meal immediately prior to receiving anesthesia will result in a full stomach, gastric volume after a few hours may not be significantly different even with longer fasting times with either fluids or solids (Table 2). In fact, an ultrasound study of healthy children (36–66 months old) found a mean gastric emptying time of 236 min—or, a little less than 4 h—after a light breakfast.^26^

**TABLE 2:** Fasting data demonstrating varying rates of gastric emptying.

Clear fluids	
Randomized clinical trial using orogastric tube^29^ 1–16 years old, *n* = 131 5 mL/kg max (up to 150 mL) of unspecified clear fluid	Gastric pH (*p* = .66): 1.43 (IQR 1.3–1.56; fasted 60 min) 1.44 (IQR 1.29–1.68; fasted 120 min) Residual volumes (*p* = .47) 0.43 mL/kg (IQR 0.21–0.84; fasted 60 min) 0.46 mL/kg (IQR 0.19–0.78; fasted 120 min) No significant difference in pH or residual gastric volume between 60 and 120 min fasting
Prospective observational study of obese and nonobese children^30^ 6–14 years old, *n* = 70 3 mL/kg 5% dextrose	Median (IQR [range]) gastric emptying time (ultrasound measurement) for: Obese children (BMI > 95th percentile) = 35 min (30.0–40.0 [25–60]) Nonobese children (BMI < 85th percentile) = 35 min (30.0–45.0 [20–60]) Median of differences 0.0, 95% CI −5.0 to 5.0; *p* = .563 Gastric volume returned to baseline within 60 min in both groups
Light meal	
MRI interventional crossover study^31^ 6–12 years old, *n* = 18	Mean residual calculated gastric volumes: 0.72 ± 0.85 mL/kg (fasted 4 h) 0.47 ± 0.25 mL/kg (fasted 6 h) Similar residual gastric volumes after 4 or 6 h fast in healthy children (*p* = .88)
Ultrasound study^26^ 33–66 months old, *n* = 30	Mean gastric antral area: 10.4 ± 3.7 cm^2^ (fasted 51 ± 31 min) 5.5 ± 2.6 cm^2^ (fasted 146 ± 33 min) Gastric antral area correlates significantly (*p* < .0001) with fasting time Calculated (regression equation gastric volume = −0.77 × fasting time + 178.9) mean gastric emptying time was 232 min for gastric volume of 0 mL

Abbreviations: BMI, body mass index; IQR, inter quartile range; MRI, magnetic resonance imaging.

With regard to clear fluids, an MRI study measuring gastric volumes 30 minutely determined a median individual t_1/2_ of 23.6 min for clear fluids (diluted raspberry syrup, 6–12 years old) with large inter‐individual variability, range of 17.9–47.8 min.^27^ It has been demonstrated that in healthy children (8–12 years old), gastric volume will return within range of baseline following 3 mL/kg of diluted raspberry syrup within 1 h, but not 7 mL/kg.^28^ Thus, the clear fluid fasting recommendation from ANZCA is for 3 mL/kg/h^15^; no specified volumes have been proposed from the other guidelines.

It may be prudent to exempt certain special populations from standard fasting recommendations due to their unpredictable gastric emptying. One of these populations includes children suffering traumatic injuries. Bricker et al.^32^ found in children aged 1–14 years with limb or facial injuries, that gastric volume aspirates remained >0.4 mL/kg in 49% of children who had fasted for more than 8 h, and 31% of children who were injured three or more hours after eating. The gastric emptying in preterm infants may also be slightly delayed compared with term infants; however, the significance of this is unclear, and so no definitive recommendations have been made in this patient group.^16^

A point of uncertainty remains with chewing gum. A meta‐analysis in 2015 by Ouanes et al.^33^ compared four RCTs, one of which was in children aged 5–17 years (the remainder were adult studies). They found chewing gum preoperatively resulted in a small but statistically significant increase in gastric volume, but no change in gastric pH.^33^ Interestingly, the included RCT in children actually showed a higher pH in children who chewed both sugarless and sugared gum (mean pH 2.19 and 2.25, respectively), compared with children who did not (mean pH 1.91).^34^ The 2023 guideline update for the American Society of Anesthesiology has recommended not delaying elective procedures in healthy adult patients who are chewing gum, though makes no recommendations for the pediatric population.^20^ As described in Table 1, both ANZCA and the recent ESAIC update recommend discarding prior to induction, as while it may not increase the risk of aspiration of gastric contents, it does pose a risk as a foreign body.^15^, ^16^ The APABGI recommends 2‐h fasting period from gum.^25^

The concern with inadequately fasted patients is the risk of aspiration. However, there appears to be a very low incidence of serious complications due to aspiration in children. One prospective multi‐center survey found 24 reported cases of aspiration from 118 371 emergency and elective cases performed over a 12‐month period.^10^ This survey ranged in ages from infant to 17 years old and American Society of Anesthesiology (ASA) classifications from 1 to 3.^10^ Of the 24 reported cases, five required ventilation, but all made a full recovery.^10^ Another single‐center retrospective audit over 13 years flagged 22 cases of pulmonary aspiration from 102 425 emergency and elective cases, with two requiring ventilation.^11^ Again no mortalities were recorded.^11^ A report using the Wake UP Safe registry found 135 cases of pulmonary aspiration from 2 440 810 emergency and elective anesthetics over 8 years.^12^ Here, two deaths were recorded; both patients suffered from significant pre‐existing comorbidities (cerebral palsy, and rhabdomyosarcoma, respectively).^12^ Eisler et al.^35^ identified 26 cases of aspiration in the 47 272 anesthetics given over 7 years, with no recorded mortality. The majority of this occurred perioperatively (23 of the 24 cases where timing could be determined from documentation), occurring at induction, during maintenance, and on emergence (9, 10 and 4 children, respectively, representing 35%, 38%, and 15%, respectively).^35^ The final case occurred in recovery.^35^ Only two of these cases were classified as ASA 1; the remaining patients were reported to have neurological, gastroenterological, or cardiorespiratory comorbidities.^35^ These cases highlight the importance of medical comorbidities in the assessment of risk of aspiration, as opposed to fasting period alone, which is also reflected in the updated ESAIC guidelines.^16^

## 3 PROLONGED FASTING

Despite national recommendations and individual institutional guidelines, it seems many children are still fasted excessively. Even in elective settings, fasting times of up to 23.5 h have been reported.^3^, ^36^ The reason behind this appears multifactorial: communication issues, theater delays, differing instructions, and parental non‐compliance (the majority of whom appear to over‐fast^37^) all contributing to excessive fasting times.^3^, ^29^ This has both psycho‐social and physiological consequences for the child (Table 3), as well as being potentially distressing for both the child and parents.

**TABLE 3:** Consequences of prolonged fasting in children.

Social	
General discomfort, irritability, and fatigue	Schmidt et al.^2^ found that children permitted liberal clear fluids until premedication (mean of 48 min pre‐induction) reported decreased thirst compared with 2 h fasted (36% and 18%, respectively), as well as higher parental satisfaction (81% vs 55%). Engelhardt et al.^3^ found 56% of children fasted for a median of 12 h reported being very hungry or “starving.” Interestingly, this study found decreased reports of hunger with increased fasting time.^3^ Al‐Robeye et al.^4^ reported 34% reporting being “hungry” or “very hungry,” and 19% reported being “thirsty” or “very thirsty.” Multiple children reported sadness and increased anxiety during the fasting period^4^
Physiological	
Hypoglycemia	Prolonged fasting (>8 h) in children <47 months and 15.5 kg resulted in hypoglycemia in 15.2% of cases, versus none in children fasting for 4 h; the difference was significant (*p* = .05).^5^ A cutoff of 40 mg/100 mL (2.2 mmol/L) was used to define hypoglycemia.^5^ Another study showed no significant difference in children <36 months (though low‐normal glucose was recorded as 4.8 ± 1.1 mmol/L [mean] with prolonged fasting, compared with 5.1 ± 1.0 mmol/L, *p* = not significant [no *p*‐value published])^6^
Ketoacidosis/metabolic acidosis without hypoglycemia	In children <36 months, deviation from the 6–4–2 fasting guidelines by more than 2 h resulted in a significant increase in ketone bodies and a lowered base excess compared with deviation for less than 2 h (mean ketones 0.8 ± 0.9 vs 0.2 ± 0.2 mmol/L [*p* < .001], and base excess −2.4 ± 3.0 vs −1.1±2.9 mmol/L [*p* < .05], respectively)^6^
Hemodynamic instability and hypovolemia	In children <36 months, increased incidence of MAP <40 mmHg on induction with prolonged fasting; MAP was also lower perioperatively in the prolonged fasting group (mean 55.2 ± 9.5 mmHg vs 50.3 ± 9.8 mmHg, *p* = .015)^7^
Insulin resistance	Develops during fasting due to decreased insulin release and increased free fatty acids; worsened during stress response of surgery due to concomitant hormone release (catecholamines, cortisol, glucagon)^8^

Abbreviation: MAP, mean arterial pressure.

A main concern is the development of insulin resistance, which results in postoperative hyperglycemia and its immediate sequalae (including increased risk of infection).^8^ Fasting tests in 167 children divided between three age ranges: 0–24 , 25–84 , and 85–216 months^38^ demonstrated that in all age groups, there was increased free fatty acids (FFA), decreased FFA/ketone body ratios, decreased insulin levels, and increased cortisol levels at the end of their fasting tests at 24 h, in keeping with the development of insulin resistance.^38^

Data from the adult population have drawn a concerning correlation between prolonged fasting resulting in increased insulin resistance, increased postoperative complications, and increased mortality. Sato et al.^39^ demonstrated in the adult cardiac surgical patient, a well‐studied cohort, that regardless of the presence of diabetes, there were increased major complications (death, cardiac failure, stroke, dialysis or severe infection) and minor infections for every 1 mg/kg/min reduction in insulin sensitivity. They also showed a 5–6× increase in major complications with postoperative decreases in insulin sensitivity of 50%.^39^ Additionally, a single regression analysis performed on several studies in Sweden, in the adult population, showed three independent predictors of prolonged postoperative hospital admission: type of surgery, blood loss, and postoperative insulin resistance.^40^ Currently, no similar studies have been done in the pediatric population.

## 4 TOLERANCE STRATEGIES

There have been several strategies exploring improving tolerance to fasting.

### 4.1 Education and encouragement

Giving children food and fluids until the latest possible time will improve the preoperative metabolic condition. This might appear fairly logical at first glance, but this has not been reflected in past audits, even taking into account the previous clear fluid fasting times of 2 h.^3^, ^36^ Therefore, a good starting point to optimizing fasting times in children is the repeated education of staff and parents regarding the institutional limits, and the encouragement of fluids and a light breakfast where appropriate.

Dennhardt et al.^7^ encouraged and educated ward staff and parents to allow children to eat until pre‐specified times, as well as introducing ongoing communication with theaters to better predict the patient's time to surgery. They also allowed children clear fluids in the morning, unless they were scheduled as the first case, with both active verbal and written reminders and encouragement.^7^ They compared this with the results from their previous study where children had the same fasting times (6 h for solids, 4 h for breast milk or formula, and 2 h for clear fluids), but only had standard written instructions.^7^ In this way, they were able to demonstrate a decrease in prolonged fasting (mean 6 vs 8.5 h), lower ketone body concentrations (mean 0.2 vs 0.8 mmol/L), more stable hemodynamics, and improved comfort.^7^ This strategy could easily be replicated in most institutions, through providing information and responding to ongoing updates, though it may increase staff workload.

### 4.2 Liberal fluids

Given the evidence for minimal harm with clear fluids up to 1 h preoperatively, the provision of liberal fluids to decrease the sensation of hunger could be further considered. There are no current studies investigating the use of clear fluids in the preoperative period to improve tolerance to fasting (carbohydrate drinks notwithstanding) as the primary outcome, though there are studies which investigate this as a secondary outcome. However, there has been research in the adult population, in a nonperioperative setting, showing the ingestion of water pre‐meal decreases the meal energy intake, and increases the feelings of fullness.^41^, ^42^

Additionally, a randomized control trial in children by Schmidt et al.,^2^ testing liberal fluids (clear fluids until premedication) against standard fasting (fluids until 2 h pre‐anesthesia), found as a secondary outcome decreased reports of thirst in those allowed liberal fluids (18% vs 36%; *p* = .030).^2^ This group also had higher parental satisfaction (81% vs 55%, *p* = .006).^2^

Further research could focus on thirst and hunger reduction as primary outcomes, especially given liberal water would be an easy and cost‐effective method of increasing feelings of satiety.

### 4.3 Carbohydrate drinks

Several studies have investigated the introduction of carbohydrate‐rich drinks given preoperatively in elective patients to prevent the development of postoperative insulin resistance. However, most of these studies have been conducted in the adult population, where the standard carbohydrate load has been set as at least 45 g given less than 4 h preoperatively.

Ready‐to‐use 12.5% carbohydrate‐rich drinks are one of the most frequently used preoperative preparations. These are classified as clear liquids, with no protein or fat.^15^ A study of 20 healthy adult volunteers found that 400 mL of a clear carbohydrate drink (50 g of carbohydrate) emptied completely from the stomach in 94 min.^43^ Therefore, they would not increase aspiration risk during anesthesia. A common regime is to give 800 mL (100 g) the night prior to surgery, and 400 mL (50 g) 2 h preoperatively.^44^ A metanalysis of 21 RCTs examining preoperative carbohydrate treatments in the adult population across multiple surgical specialties (general surgery, orthopedics, cardiac) has found beneficial effects including reduced thirst, hunger, anxiety, and malaise; improved preservation of insulin sensitivity; decreased hyperglycemia; improved protein metabolism; and decreased postoperative nausea and vomiting.^44^ It may also reduce the length of hospital admission.^44^

The use of carbohydrate drinks in the pediatric population has been less well investigated. From the limited available evidence, they appear safe in children, are well tolerated, and do not seem to increase aspiration risk during anesthesia; they are however not yet used routinely in the preoperative preparation of children.^45^, ^46^ The varied metabolic stress response in children means the standard carbohydrate‐rich drinks may not result in the same outcomes as noted in the adult population; but they may confer the same benefit of reduced insulin resistance.^45^

In a prospective study, 86 children aged 1–11 years were given 15 mL/kg (<3 years) or 10 mL/kg (>3 years) of 12.8% carbohydrate drink 2 h preoperatively.^46^ Ultrasound assessment of gastric antrum area, used as a surrogate for gastric volume, was performed pre‐drink after an 8 h fast, and pre‐induction 2 h postdrink.^46^ The drinks were well tolerated, with only three children declining to ingest the drink and three children reporting nausea or vomiting postoperatively (from 79, after excluding children who did not want to ingest the drink, and those who had their surgeries delayed).^46^ Gastric antrum area was found to actually decrease after the carbohydrate drink (mean 2.09 ± 0.97 cm^2^ to 1.85 ± 0.94 cm^2^ [*p* = .01]), reflecting a decrease in gastric volume.^46^ Additionally, there were no reported cases of aspiration.^46^

A RCT of 20 children aged 4–17 years with ASA I or II classifications, compared a 12.6% carbohydrate solution given the evening prior and again 2 h preoperatively, against a standard fast.^45^ They found these drinks to be well tolerated and did not report any cases of aspiration in the small cohort.^45^ They also found that insulin resistance (calculated using the Homeostatic Model Assessment for Insulin Resistance [HOMA‐IR] equation) was higher in the standard fast group (2.0 [0.43–7.55] vs 0.62 [0.37–2.22], *p* = .03).^45^

### 4.4 Glutamine drinks

In addition to carbohydrate‐rich drinks, there have been drinks developed with further additions such as glutamine and antioxidants, aimed at improving metabolic endurance during fasting.^43^ Glutamine, which may improve immune function and gastrointestinal perfusion, and antioxidants, which may improve morbidity and mortality in critically ill patients, might therefore provide additional benefits beyond carbohydrates alone.^43^ Due to the nutrient load however, these drinks should be given 3–4 h preoperatively.^43^ Further research into these supplements in the adult population is pending, while recommendations regarding clinical efficacy and general use have not yet been made. These drinks have not been studied in the pediatric population.^11^

### 4.5 Chewing gum

There have been a few studies in the adult population which have shown that the act of chewing gum increases satiety. Melanson et al.^47^ found in a randomized crossover study of healthy adults that after a standard fast and controlled breakfast shake, adults who chewed gum had decreased hunger ratings and less caloric intake at lunch, compared with those who did not chew gum. Xu et al.^48^ found in a randomized crossover study of healthy adult males that after an overnight fast, those chewing gum reported increased satiety, as well as a slower reduction of GLP‐1 levels (a hormone implicated in satiety), compared with those who did not chew gum. This has been less well studied in children. In addition, the risk of gum becoming an airway foreign body if not removed preoperatively must be considered.

## 5 CONCLUSION

Shortened fluid fasting periods have been shown to be safe, with multiple international societies updating their recommendations to reflect the current evidence. Even in instances of fluid aspiration, no severe sequalae have been reported in children. However, children are still being fasted for unnecessarily long periods preoperatively, resulting in both patient and parental distress, as well as increasing adverse clinical outcomes. More work is required to match hospital fasting times to published guidelines. While some pathways have been explored to improve the tolerance for fasting, for example, education or carbohydrate‐rich drinks, the evidence remains limited in pediatric patients. This invites further exploration in the realm of preoperative innovations to reduce the distressing symptoms of hunger and thirst.

**Key learning points**

- There have however been increasing concerns with regard to the high incidence of prolonged fasting in children, and the subsequent psycho‐social distress and physiological consequences that may ensue.
- Pediatric fasting guidelines have recently been updated in multiple countries to reflect the latest evidence for gastric emptying, and the minimal morbidity and mortality data reflected in pediatric patients who do aspirate.
- Multiple fasting strategies exist in the literature, though these have mostly been studied and implemented in the adult population.

## FUNDING INFORMATION

BSvUS is part funded by the Stan Perron Charitable Foundation and through a National Health and Medical Research Council Investigator Grant (2009322).

## CONFLICT OF INTEREST STATEMENT

Britta S. von Ungern‐Sternberg is a section editor for Pediatric Anesthesia.

## Data Availability

The data that support the findings of this study are available on request from the corresponding author. The data are not publicly available due to privacy or ethical restrictions.
